# Dietary exposure to an environmental toxin triggers neurofibrillary tangles and amyloid deposits in the brain

**DOI:** 10.1098/rspb.2015.2397

**Published:** 2016-01-27

**Authors:** Paul Alan Cox, David A. Davis, Deborah C. Mash, James S. Metcalf, Sandra Anne Banack

**Affiliations:** 1 Institute for Ethnomedicine, Jackson Hole, WY, USA; 2 Department of Neurology, University of Miami, Miller School of Medicine, Miami, FL, USA

**Keywords:** Alzheimer's, amyotrophic lateral sclerosis, l-serine, cyanobacteria, BMAA, tau

## Abstract

Neurofibrillary tangles (NFT) and β-amyloid plaques are the neurological hallmarks of both Alzheimer's disease and an unusual paralytic illness suffered by Chamorro villagers on the Pacific island of Guam. Many Chamorros with the disease suffer dementia, and in some villages one-quarter of the adults perished from the disease. Like Alzheimer's, the causal factors of Guamanian amyotrophic lateral sclerosis/parkinsonism dementia complex (ALS/PDC) are poorly understood. In replicated experiments, we found that chronic dietary exposure to a cyanobacterial toxin present in the traditional Chamorro diet, β-*N*-methylamino-l-alanine (BMAA), triggers the formation of both NFT and β-amyloid deposits similar in structure and density to those found in brain tissues of Chamorros who died with ALS/PDC. Vervets (*Chlorocebus sabaeus*) fed for 140 days with BMAA-dosed fruit developed NFT and sparse β-amyloid deposits in the brain. Co-administration of the dietary amino acid l-serine with l-BMAA significantly reduced the density of NFT. These findings indicate that while chronic exposure to the environmental toxin BMAA can trigger neurodegeneration in vulnerable individuals, increasing the amount of l-serine in the diet can reduce the risk.

## 1. Introduction

### (a) Toxins and neurodegenerative illness

The relationship between environmental toxins and neurological disease has been of interest since residents of Minamata Bay, Japan, were sickened by chronic dietary exposure to methyl-mercury-laden fish. Parkinson's disease (PD) has been linked to rotenone or paraquat exposures in agricultural workers [1]. PD also was diagnosed in ‘frozen addicts’, users of a recreational drug contaminated with 1-methyl-4-phenyl-1,2,3,6-tetrahydropyridine [2]. Exposures to pesticides, metals, solvents and certain types of volatile anaesthetics have additionally been linked to PD, while exposures to lead, mercury and pesticides have been suggested as risk factors for amyotrophic lateral sclerosis (ALS) [1]. However, the role of naturally occurring environmental toxins in progressive neurodegenerative disease has not been extensively studied.

### (b) A paralytic disease among Pacific Islanders

In the 1950s, US Army physicians described a puzzling ALS-like disease among the indigenous Chamorro villagers of Guam [3]. In the 1960s, amyotrophic lateral sclerosis/Parkinsonism dementia complex (ALS/PDC) was described based on histopathology and clinical symptoms which resemble aspects of Alzheimer's disease (AD), ALS and PD. Neurofibrillary tangles (NFT) in the brains of individuals with ALS/PDC have similar immunohistology and structure as those found in the brains of AD patients but are biochemically and regionally more heterogeneous [4,5]. Many afflicted villagers suffered from dementia. Histopathology of Chamorros who died prior to manifesting clinical symptoms of ALS/PDC suggests that depositions in the brain pre-dated clinical onset [4]. No clear pattern of inheritance for the disease has been ascertained [6]. Since even outsiders who adopted a Chamorro lifestyle experienced an increased risk of illness [7], a common environmental exposure seemed likely. Determining the nature of the toxin, however, was difficult due to a significant delay between exposure and clinical symptoms, extending years or even decades [8].

### (c) BMAA: a neurotoxic amino acid in cycad seeds

In the 1960s, consumption of flour made from the gametophyte of cycad seeds (*Cycas micronesica* Hill) was proposed as a cause of the disease. Interest increased when a novel neurotoxic amino acid, l-BMAA, was isolated from cycad seeds by Bell [9]. In the 1980s, BMAA fed to macaques was found to cause acute neurological symptoms [10], a finding that was discounted when it was argued that an equivalent human dose would require the consumption of unreasonable amounts of cycad seed flour [11]. BMAA was subsequently identified as a cyanobacterial product [12]. The toxin is biomagnified in flying foxes, which are eaten by Chamorros [13]. Equally important was the discovery that a majority of BMAA in cycad seeds binds to proteins and cannot be released by washing with water, but only on hydrolysis, suggesting that BMAA doses ingested by the Chamorros had been previously underestimated [14,15]. Evidence continued to build for the link between BMAA and neurodegenerative disease with respect to cyanobacterial exposure and epidemiology [15–20].

A key missing puzzle piece has been experimental evidence that chronic dietary exposure to BMAA triggers neuropathological changes consistent with ALS/PDC, which presumably should occur prior to the onset of clinical symptoms. It is now known that *in vivo* BMAA exposure generates fibril formation and cognitive deficits in rodents [21], although some earlier animal studies that focused on acute rather than chronic exposure were inconclusive [22]. This finding suggests that chronic BMAA exposure more closely models early disease.

### (d) BMAA in the Chamorro diet

BMAA is produced by symbiotic cyanobacteria of the genus *Nostoc* harboured in specialized cycad roots emergent in the leaf litter above the soil. BMAA accumulates in the gametophytes of cycad seeds, which, after washing, are used by villagers to prepare tortilla flour, dumplings and to thicken soups and stews. Animals, including flying foxes, feral deer and pigs that feed on cycad seeds, which in turn are consumed by villagers, also accumulate BMAA in their tissues [13]. BMAA is biomagnified up to 10 000-fold from its production by cyanobacteria to its concentration in volant mammals [12,13,15].

### (e) BMAA exposures beyond Guam

Diverse taxa of cyanobacteria produce BMAA [23,24], which is biomagnified in some marine ecosystems, accumulating in sharks, bottom-dwelling fish and shellfish. BMAA also occurs in cyanobacterial soil crusts [25]. BMAA exposure through inhalation of desert dust has been suggested as triggering the increased incidence of ALS a decade subsequent to the deployment of military personnel in Operation Desert Storm [26]. Similarly, inhalation of aerosolized BMAA from wave break has been proposed to explain the increased risk of ALS in individuals who live near lakes with persistent cyanobacterial blooms [18,19]. Exposure through ingestion of drinking water has not been ruled out [27]. Maternal exposures to BMAA may also increase the risk of ALS in neonates later in their life [20,21].

### (f) Mechanisms of BMAA-induced neurodegeneration

Through activation of metabotropic glutamate receptors such as mGluR5 [28] or ionotropic glutamate receptors including the *N*-methyl-d-aspartate receptor, kainate or the *α*-amino-3-hydroxy-5-methyl-4-isoxazolepropionic acid receptor, BMAA selectively kills subpopulations of NADPH-diaphorase-positive motor neurons [29]. It is toxic to glial cells [30] and causes motor neuron damage and astrogliosis in the ventral horn [31]. BMAA potentiates different neurotoxic insults including methyl mercury [32], which co-occurs in some fish. BMAA rapidly crosses the blood–brain barrier, where it is captured by the central nervous system (CNS) in a time period consistent with protein misincorporation [33]. BMAA can be mistaken by cellular machinery for l-serine and be misincorporated into proteins, leading to protein misfolding, aggregation and subsequent apoptosis [34]. Misincorporation of BMAA into proteins has been proposed as a mechanism for bioaccumulation as well as a mechanism for slow release of BMAA within the CNS over years depending on rates of protein turnover [15]. Misincorporation of even the 20 canonical amino acids at error rates as low as 1/10 000 can lead to neurodegeneration in laboratory animals [35]. BMAA exposure results in hyperphosphorylated tau, possibly by decreasing activity of protein phosphatase 2A (PP2A) through activation of the mGluR5 receptor and subsequent dissociation of the catalytic subunit PP2Ac [36]. In Chamorro ALS/PDC brains, PP2A activity is significantly decreased, resulting in a significant increase in hyperphosphorylated tau [36].

### (g) Producing an animal model of BMAA-induced neuropathology

The occurrence of BMAA in post-mortem brain tissues of Chamorro ALS/PDC patients but generally not in non-Chamorro control patients suggests that chronic dietary exposure to BMAA is an environmental risk factor for ALS/PDC [12,15,37]. To satisfy Koch's postulates of disease causation [38], it is necessary to show that chronic exposure to BMAA causes healthy individuals to develop neurodegenerative disease and that BMAA can be re-isolated from those individuals in which neurodegeneration has been induced.

NFT and β-amyloid deposits have not both been produced in human neuronal cell culture, so *in vivo* experiments are necessary. However, no animal species other than humans is known to develop ALS/PDC or AD. Furthermore, NFT and β-amyloid deposits have not both previously been produced in any single animal model, with the exception of a triple transgenic mouse model [39] in which the structure and density of the NFT significantly differ from the human condition (K. Iqbal 2015, personal communication). Some non-human primates including squirrel monkeys, chimpanzees, gorillas and orangutans of great age as well as lemurs develop senile plaques that are immunopositive for β-amyloid, and a single 41-year-old chimpanzee was found to produce paired helical tau filaments [40]. Vervets are known to accumulate vascular β-amyloid deposits with age, but not NFT and other tau inclusions [41]. We therefore decided to chronically expose vervets to BMAA for an extended period and to examine their brain tissues for tau inclusions and amyloid deposition consistent with ALS/PDC pathology. Since matching the duration of chronic exposure to the years—even decades—required for Chamorros to develop ALS/PDC [8] is unfeasible, we shortened chronic exposure to BMAA to 140 days. Since l-serine has been found to prevent misincorporation of BMAA and apoptosis in human neuronal cell culture [34], we added a cohort of vervets which daily received equal amounts of BMAA and l-serine. Finally, to increase statistical rigour, we replicated the first experiment. We used an oral dose (210 mg kg^−1^ d^−1^) that previous investigators found using gavage could be tolerated by macaques [10] and in the second experiment added a cohort of vervets with a 10-fold dose reduction (21 mg kg^−1^ d^−1^) to produce a cumulative BMAA exposure closer to total lifetime Chamorro exposure.

## 2. Material and methods

### (a) *In vivo* studies

The vervets studied in this report were housed in groups in large outdoor enclosures at the Behavioural Science Foundation (BSF) in St Kitts, West Indies. The BSF is a fully accredited biomedical research facility with approvals from the Canadian Council on Animal Care. The animal use protocol was approved by the Institutional Animal Care and Use Committee (IACUC) of BSF and McGill University (Quebec, Canada). The vervet low-protein diet was supplemented with fruit dosed with l-BMAA or other test substances. Doses were prepared at the Institute for Ethnomedicine, Jackson Hole, using a Mettler Toledo balance with a Quantos automated powder dispensing module at a tolerance of ±0.1% of target dose. A small cavity was made in each piece of fruit, and the test substance was placed inside. In the first experiment, 16 juvenile vervets were presented daily with a dosed piece of fruit, approximating a 210 mg kg^−1^ d^−1^ dose based on average weight (3.1 kg) of the vervets. One cohort of four was fed daily for 140 days a piece of fruit containing 651 mg of l-BMAA, a second cohort was fed fruit with 651 mg of l-serine, a third cohort was fed fruit dosed with 651 mg of l-BMAA plus 651 mg of l-serine, and a fourth control cohort received a piece of fruit dosed with 651 mg of rice flour as a placebo.

For both the original experiment on the younger vervets and the replication experiment on the adult vervets, 14 regions of each vervet's brain were investigated for neuropathology with immunostaining for tau AT8 and β-amyloid 1–42. Three serial sections were studied on a 3 × 4 grid with 100× magnification. In the replication experiment, cohorts of eight adult vervets were fed dosed fruit for 140 days. These 7-year-old vervets, which were colony-born, were somewhat larger than the younger vervets in the first experiment, so to approximate a 210 mg kg^−1^ d^−1^ dose for these larger animals, the l-BMAA dose was increased from the first experiment in order to adjust for weight. A cohort of vervets was added in which the l-BMAA dose was reduced 10-fold to approximate 21 mg kg^−1^ d^−1^ to be closer to a lifetime Chamorro exposure. Thus in the replication experiment one cohort of eight vervets received daily 987 mg of l-BMAA, a second cohort received 98.7 mg of l-BMAA, a third cohort received 987 mg of l-BMAA and 987 mg of l-serine, and a fourth control cohort received 987 mg of rice flour. Periodic blood serum and cerebral spinal fluid (CSF) samples were taken under ketamine anaesthesia to confirm BMAA exposures in the vervets and absence of BMAA exposure in the controls.

### (b) Neuropathology

In both experiments, one hemisphere of each vervet brain was frozen. The other hemisphere was immersion fixed in buffered formalin for histopathology. This hemisphere was freeze-sectioned at 40 µm and an adjacent series of coronal sections were processed with antibody stains using the MultiBrain Services of NeuroScience Associates (Tennessee, USA). In both experiments, adjacent sections were stained with AT8 immunohistochemistry (IHC) stain with a thionine Nissl counterstain for hyperphosphorylated tau and β-amyloid (1–42) IHC stain for β-amyloid deposits. In the first experiment, NFT (100× magnification) and β-amyloid deposits (10× magnification) were identified from blinded review of the stained sections and were quantified using manual counts in three sections in series from non-overlapping brain regions. In the second experiment, stained sections were examined using automated images prepared with a TissueScope LE (Huron Digital Pathology, Ontario, Canada). Stained serial sections were digitally scanned at 20× using a 350 µm^2^ grid for NFT and β-amyloid deposits. Thioflavine-S with a thionine Nissl counterstain was used to confirm the presence of NFT and plaques. The regions of interest (ROI) for each case were initially drawn on the Nissl section and the ROI was mapped to the immunostained slides. The ROI was marked with an array tool to identify regional boundaries of the amygdala, hippocampus, entorhinal, frontal, temporal, motor, occipital and cingulate cortices. Digital images were measured using NIH Image J64 software (1.44) converted from RGB colour to 8-bit followed by applying a threshold to eliminate non-specific background staining. After threshold correction, the images were converted to binary allowing for quantification of pathological features detected above background. The high-contrast images were highly suited for digital quantification of pixel counts. Representative sections were examined in parallel to validate the digital measurements by comparison to manually derived β-amyloid deposits and NFT counts [42].

### (c) Analytical chemistry

Blinded samples of brain tissue, blood serum and CSF were analysed for BMAA content using triple quadrupole tandem mass spectrometry (LC-MS/MS) with a precolumn 6-aminoquinolyl-*N*-hydroxysuccinimidyl carbamate (AQC) derivatization using the validated method determined by the Association of Analytical Communities AOAC International [43]. Negative controls included matrix blanks from control vervets with no detectable BMAA, AQC-derivatized blanks, internal standards and solvent blanks (HCl, TCA). Product ion analysis of BMAA used *m/z* 459 as the precursor ion for collision-induced dissociation (CID) and two-step mass filtering was performed during selective reaction monitoring of BMAA after CID in the second quadrupole, monitoring the following transitions: *m/z* 459 to 119 CE 25 eV; 459 to 289 CE 23 eV; 459 to 171 CE 45 eV. The resultant product ions were detected after passing the third quadrupole and their relative abundances were quantified. BMAA was analytically distinguished from its isomers using *m/z* 459 to 188 CE 38 eV (2,4-diaminobutyric acid); 459 to 214 CE 35 eV (*N*-(2-aminoethyl)glycine); 459 to 258 CE 36 eV (BMAA) [44,45]. Double ionized AQC-derivatized BMAA was also monitored with a precursor ion of *m/z* 230 and a product ion of 171 CE 27 eV. Additionally, the following amino acids were monitored: single derivatized lysine *m/z* 317, double derivatized lysine *m/z* 487, leucine *m/z* 302, serine *m/z* 276 and an internal standard (β-*N*-methyl-d3-amino-DL-alanine-^15^N_2_) with a precursor ion of *m/z* 464, and product ions *m/z* 124 CE 25 eV, 171 CE 45 eV, 259 CE 36 eV and 294 CE 23 eV. BMAA tissue concentrations were determined relative to concentration curves run daily in spiked matrix samples from a control animal using β-*N*-methyl-d3-amino-DL-alanine-^15^N_2_ as an internal standard. Sample preparation techniques and complete analytical methods appear in the electronic supplementary material.

### (d) Statistical analysis

Because of the small sample size inherent in the experimental design, and to avoid assumptions of normal distribution of resultant data, we used non-parametric methods to compare medians. To determine if chronic dietary exposure to BMAA results in a greater density of NFT, the hypotheses *H*_0_ = there is no difference in median NFT density between treatment groups and *H*_1_ = there is a difference in median NFT density between treatment groups were evaluated with a Kruskal–Wallis *H*-test, a non-parametric analogue of an analysis of variance. A Jonckheere–Terpstra trend test was used to test the hypotheses *H*_0_ = median NFT density is independent of BMAA dose versus *H*_1_ = median NFT density increases with BMAA dose. Different hypotheses comparing median amounts of BMAA per cohort treatment group were evaluated with a Kruskal–Wallis *H*-test: *H*_0_ = there is no difference in median BMAA concentrations between treatment groups within plasma, brain or CSF, and *H*_1_ = there is a difference between median BMAA concentrations between treatment groups in plasma, brain or CSF. (In this case, medians of protein-bound BMAA were used for plasma and brain samples, while medians of total BMAA content were used for the CSF; see methods in electronic supplementary material.) Other hypotheses evaluated with a Kruskal–Wallis *H*-test were *H*_0_ = there is no difference in the median ratios of protein to total BMAA concentrations between treatment groups in plasma or brain, and *H*_1_ = there is a difference in the median ratios of protein to total BMAA concentrations between treatment groups in plasma or brain. To determine if chronic dietary exposure to BMAA is related to the presence of β-amyloid deposits, two alternative hypotheses were evaluated with a *χ*^2^ test: *H*_0_ = there is no difference between treatment types on the number of vervets that develop β-amyloid deposits, and *H*_1_ = there is a difference between treatment types on the number of vervets that develop β-amyloid deposits. Spearman's rank correlation coefficients were calculated to evaluate *H*_0_ = there is no relationship between protein-bound or protein-bound/total ratio of BMAA concentrations in the brain and NFT counts, and *H*_1_ = there is a relationship between protein-bound or protein-bound/total ratio of BMAA concentrations in the brain and NFT counts.

## 3. Results

**Figure 1.**
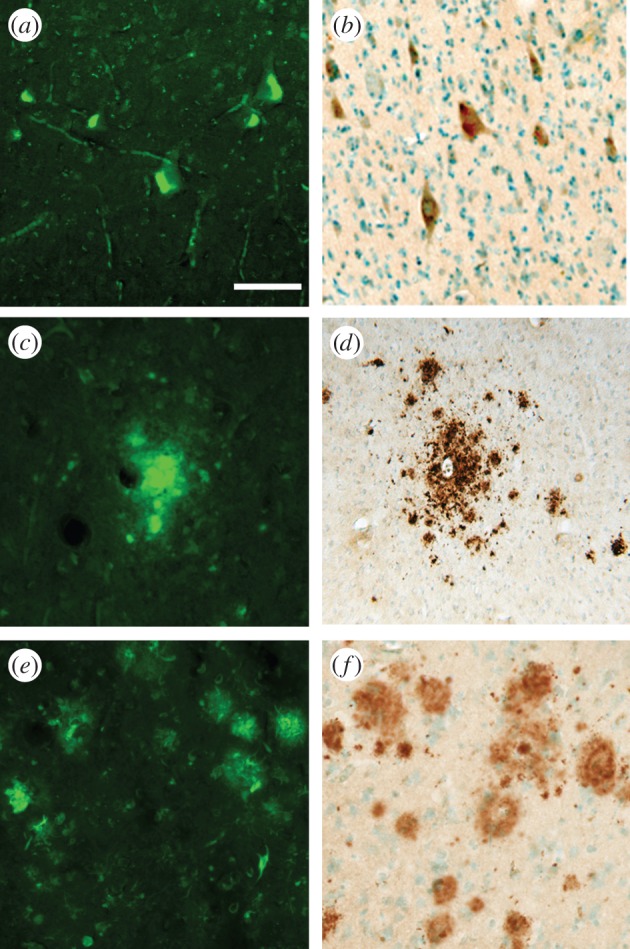
Neuropathology of vervet brain tissue with chronic dietary BMAA exposures; a comparison of thioflavine-S and β-amyloid (1–42) immunoreactivity. (*a*) Thioflavine-S stained cells and neuropil threads in the motor cortex; scale bar, 150 µm. (*b*) Intraneuronal β-amyloid accumulation in neurons in motor cortex. (*c*) Vervet extracellular thioflavine-S deposits in the frontal cortex. (*d*) Localized β-amyloid immunostained neocortical deposits in vervet brains. (*e*) Thioflavine-S positive senile plaques and tangles in human AD temporal cortex. (*f*) β-amyloid senile plaques in human temporal cortex of AD patient (86-year-old male; 400× magnification). Human brain sections from AD patients were run as reference controls.

**Figure 2.**
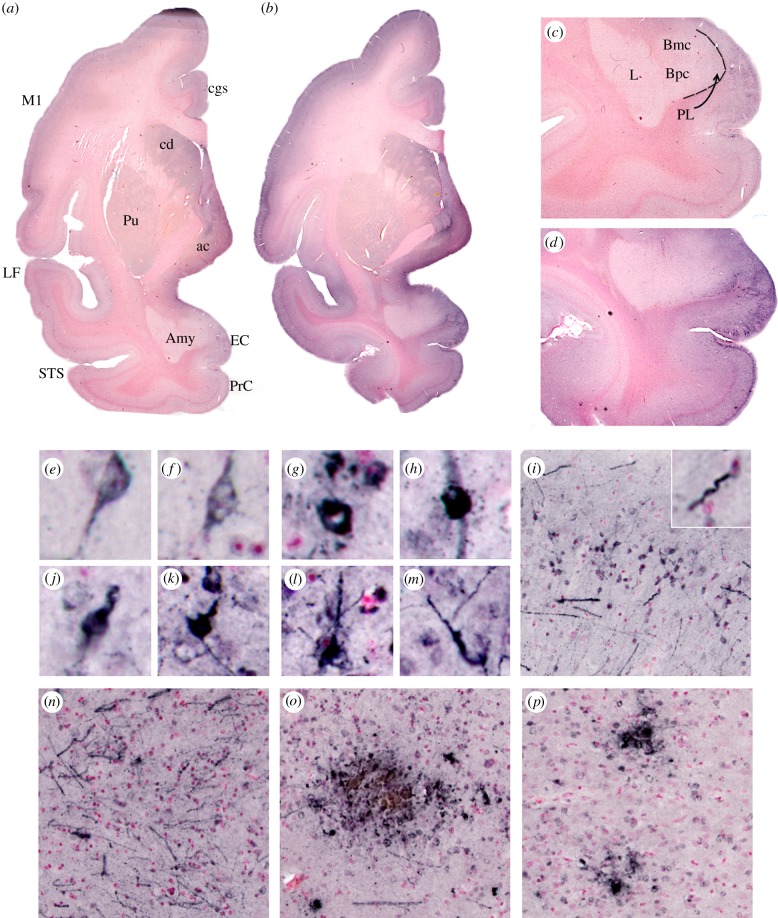
Microscopic pathology of chronic dietary l-BMAA exposures in vervets. Representative low-power images (5× magnification) of hyperphosphorylated tau (AT8) immunostained coronal hemisections from control (*a*,*c*) and l-BMAA-fed vervets (*b*,*d*). AT8 immunostaining is seen in the amygdala (Amy), entorhinal (EC), perirhinal (PrC), primary motor (M1) and temporal cortices of l-BMAA-fed vervets. Higher-power images show predominant tau AT8 staining in superficial cortical layers II and III with more robust staining over the entorhinal and perirhinal cortices (25× magnification) (*d*). Microscopic images (original magnification ×120) show NFT in vervets fed l-BMAA. Tangle-like tau aggregates are seen in the temporal gyrus (*e*,*f*). Dense intracellular tau immunolabelling (*g*–*i*) and extracellular deposits (*j*,*k*) were seen in the parahippocampal gyrus. Abundant neuropil threads, tangles and dystrophic neuronal processes are observed in layers II and III of the perirhinal cortex (*I*, high-power images shown in *l*,*m*) and the paralaminar nucleus of the amygdala (*n*). Tau plaques were seen in l-BMAA-fed vervets ranging from large and diffuse (*o*) to small dense aggregates (*p*). ac, anterior commissure; Amy, amygdala; Bmc, basal nucleus of the amygdala, magnocellular region; Bpc, basal nucleus of the amygdala, parvicellular subdivision; Cd, caudate; cgs, cingulate gyrus sulcus; EC, entorhinal cortex; L, lateral nucleus of the amygdala; LF, lateral fissure; M1, primary motor cortex; PrC, perirhinal cortex; PL, paralaminar nucleus; Pu, putamen; STS, superior temporal sulcus.

**Figure 3.**
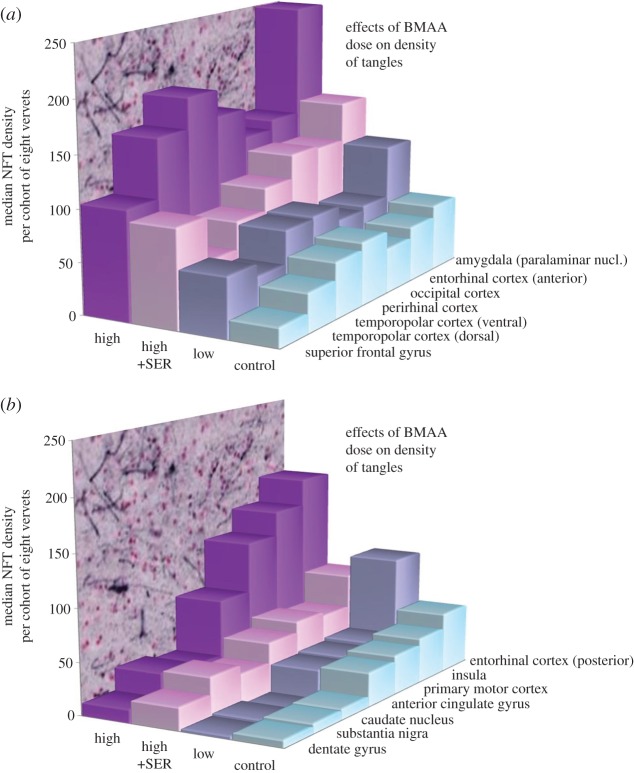
Median counts for density of AT8 IHC positive staining inclusions plus NFT per brain area by treatment type. Each horizontal surface represents the median of an eight-vervet cohort statistically significant for dose using the Jonckheere–Terpstra trend test. (*a*) Brain regions in which AT8 IHC positive density counts from the 210 mg kg^−1^ d^−1^ BMAA treatment are greatest compared with other treatment types. (*b*) Brain regions in which AT8 IHC positive density counts from the 210 mg kg^−1^ d^−1^ BMAA plus 210 mg kg^−1^d^−1^ l-serine (high+SER) treatment is less than low-dose (low) BMAA (entorhinal cortex posterior) or in which low-dose NFT density is similar to controls (all other brain areas).

In the first experiment, AT8-positive tangles and neuronal processes, as well as sparse β-amyloid plaque-like deposits, were observed in brain tissues of the l-BMAA-dosed vervets. AT8-positive NFT were observed in the perirhinal and entorhinal cortices, amygdala (paralaminar nucleus), motor cortex, frontal cortex, temporopolar cortex and occipital cortex of the BMAA-fed animals (figures 1–3). In contrast, no AT8 immunopositive inclusions were visualized in the hippocampus (CA1 or dentate gyrus). Sparse immunopositive β-amyloid deposits were observed primarily in the frontal, temporal and motor cortices. In the first experiment, the l-serine treated cohort and the control cohort of four vervets were generally negative for tau AT8 and β-amyloid 1–42 neuropathology, while there was an 80–90% reduction of NFT and plaques in the cohort fed equal amounts of l-BMAA and l-serine; these results will be published elsewhere.

**Table 1.** Median AT8 tau NFT quantification in brain tissues of vervets. Each treatment cohort consisted of eight vervets.

anatomical regions	placebo	low dose	high dose	l-serine + BMAA
superior frontal gyrus	19	58	106	95
anterior cingulate gyrus	32	28	83	48
insula	43	33	153	54
temporopolar cortex (dorsal)	38	46	162	53
temporopolar cortex (ventral)	54	77	191	79
entorhinal cortex (anterior)	53	69	145	121
entorhinal cortex (posterior)	57	106	178	85
perirhinal cortex	61	79	165	101
occipital cortex	43	65	136	124
primary motor cortex	37	30	130	58
paralaminar nuc. of the amygdala	64	118	250	158
dentate gyrus	7	3	13	22
caudate nucleus	10	1	30	29
substantia nigra	9	5	35	38

In the replication experiment, chronic l-BMAA exposures for 140 days again led to hyperphosphorylated tau deposits and NFT formation in all BMAA-fed vervets (figure 2). Median NFT density differed significantly between treatment groups (Kruskal–Wallis *H* statistic = 16.4, *p* < 0.001). Furthermore, there was a clear dose relationship between chronic dietary exposure to l-BMAA and density of NFT (Jonckheere–Terpstra trend test, *Z* = 4.4, *p* < 0.00001). NFT were abundant in vervets with chronic dietary exposure to BMAA in the superior frontal, temporopolar (dorsal and ventral), perirhinal, occipital and entorhinal (anterior and posterior) cortices, and in the amygdala (figure 3). In these brain areas, there was a highly significant dose relationship between increasing dietary exposure to l-BMAA and NFT density (Jonckheere–Terpstra trend test, *Z*-scores for the 14 brain regions range between 3.13 and 4.87, *p* < 0.001–0.00001; figure 3). The regional differences in NFT and β-amyloid deposit counts in the brain areas examined were profound. For example, in the occipital cortex, other than controls, vervets in the low-dose treatment had the lowest median count (65) NFT density, while the median density (136) of the high-dose BMAA cohort was more than twice the low-dose NFT density. Co-administration of l-serine with high-dose BMAA significantly reduced median NFT density (124). This reduction in NFT induced by l-serine occurred in all measured areas of the brain. Supplementing the diet with l-serine resulted in more than a 50% NFT reduction in median NFT densities within five brain regions: temporal (dorsal and ventral), primary motor, entorhinal (posterior) and insula cortices. In the perirhinal cortex, amygdala and anterior cingulate gyrus, l-serine reduced NFT densities more than 35%.

A highly significant (*p* < 0.00001) dose relationship between chronic dietary exposure to l-BMAA and NFT density was also found in other brain regions, but no profound differences in NFT densities were found between the low-dose and control cohorts in these regions which included the dentate gyrus, substantia nigra, caudate nucleus, anterior cingulate gyrus, primary motor cortex and the insula cortex (figure 3*b*). Finally, dose relationships by treatment group were also significant in the entorhinal cortex, but this brain area differed from the others in that co-administration of L-serine led to an NFT density not only lower than high-dose BMAA, but also lower than low-dose BMAA (figure 3*b*).

Chronic dietary exposure to BMAA significantly increased the likelihood of a vervet developing β-amyloid deposits (*χ*^2^ = 15, *p* < 0.01). One of the eight low-dose BMAA vervets, three of the eight high-dose BMAA vervets and two of the eight high-dose BMAA plus L-serine vervets had β-amyloid deposits. These β-amyloid deposits were diffuse and sparse in distribution (figure 1). β-amyloid deposits were not found in any of the control vervets.

The relationship between NFT counts and measured concentrations of BMAA in the occipital cortex was also of interest. BMAA could not be detected in control vervets or baseline samples using LC-MS/MS. Protein-bound BMAA occurred in brain tissues of individual l-BMAA-fed vervets at concentrations between 0.24 and 2.2 µg mg^−1^ (see electronic supplementary material), similar to Chamorro ALS/PDC brain tissues (median = 0.6 µg mg^−1^, range = 0.2–1.2 µg mg^−1^) [46], and was detected in blood plasma and CSF. Even within the low-dose cohorts, protein-bound BMAA within vervet brain tissues (0.24–0.78 µg mg^−1^) reached concentrations consistent with the Guam disease.

There was no significant difference in protein-bound BMAA concentrations in blood plasma between treatment groups, but there were significant differences for BMAA concentrations for brain and CSF samples (Kruskal–Wallis *H* statistics (corrected for ties) of 8.69 and 9.09 (*p* < 0.05). There was no significant difference in the ratio of protein to total BMAA concentrations in brain, but there was in blood plasma (*H* statistic = 13.24, *p* < 0.01). Finally, no significant relationship was found between protein-bound BMAA and NFT density as well as in the protein-bound/total BMAA ratio and NFT density in vervet brains as determined by Spearman's rank correlation coefficients.

## 4. Discussion

### (a) l-BMAA triggers neuropathology

Chronic dietary exposure to l-BMAA results in the formation of NFT and β-amyloid deposits in a clear dose relationship. Other protein inclusions similar to those found in brain tissues from Chamorros who died with ALS/PDC were also found. Chronic dietary exposure to l-BMAA triggered tauopathies in all BMAA-dosed vervets including those at the low-dose treatment but NFT densities varied between brain regions. Concentrations of BMAA in vervet brains fell within the range measured in post-mortem brain tissues of Chamorros who died with ALS/PDC confirming BMAA exposures in the vervets that are clinically relevant. Furthermore, the regional densities of NFT are similar in both the Chamorros and l-BMAA-fed vervets [47,48].

There was a significant dose relationship between BMAA and NFT density in all affected regions of the brains (figure 3). The distribution of NFT and their relationship to dose exposure in the temporal lobe is similar to Braak 1 early stage AD pathology [49] (figure 3). Consistent with the neuropathology of preclinical AD, no profound clinical symptoms were observed in any of these vervets in the two experiments. Specific immunological methods (AT8) permit evaluation of neuronal changes before the actual formation of NFT and neuropil threads (figure 2). In vervets with chronic dietary exposure to BMAA, we observed changes in the transentorhinal region of the temporal lobe, but none in Ammon's horn of the hippocampus. Extensively distributed NFT formations with gliosis characterize ALS/PDC. Dementia in these cases is attributable to tangles and neuronal dropout in the neocortex, resembling the pattern reported for AD, but far more widely distributed [4]. NFT in ALS/PDC brain tissues stain positively with antibodies to hyperphosphorylated tau protein [5]. Thus, the distribution of AT8-positive tangles following chronic dietary BMAA exposure in vervets is similar to the histopathology reported previously in ALS/PDC.

The paucity of clinical symptoms in the BMAA-fed vervets corresponds to the finding of NFT in 5/29 asymptomatic Chamorro patients who died without ALS or PD being recognized clinically [4]. Furthermore, β-amyloid plaques have been detected in human ALS/PDC patients who remain cognitively intact [50]. The fact that BMAA-dosed vervets produced NFT and rare β-amyloid deposits in both experiments supports the theory that BMAA in the traditional diet is a cause of the Chamorro disease.

### (b) Neurofibrillary tangles and β-amyloid deposit formation

**Figure 4.**
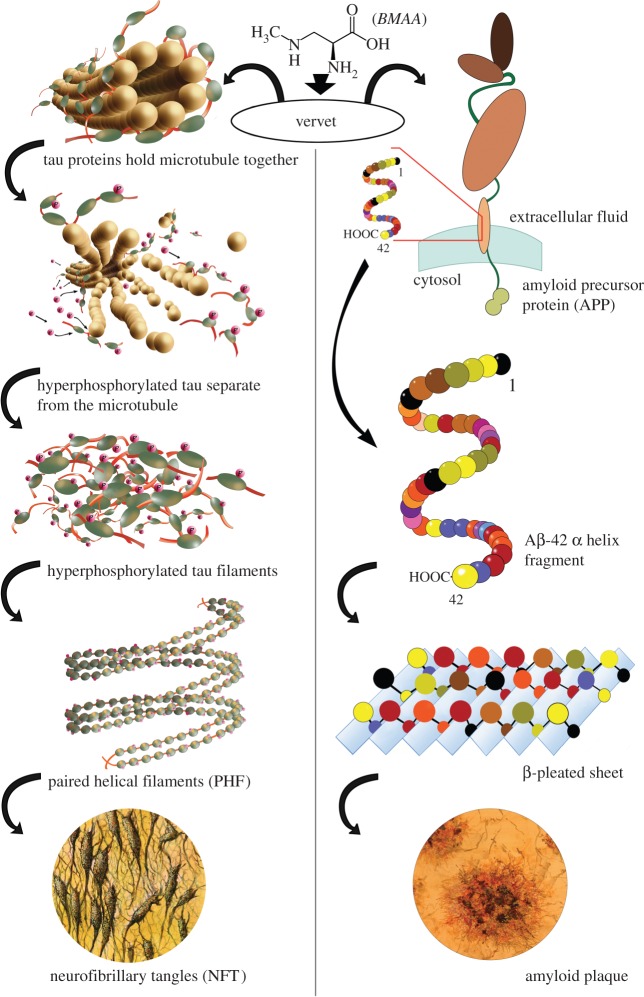
Theoretical pathways of development of ALS/PDC and AD neuropathology from chronic dietary BMAA exposure. (*a*) Tau proteins which bind microtubules become hyperphosphorylated, leading to dissociation of hyperphosphorylated tau fragments. These form paired helical filaments, leading to the formation of neurofibrillary tangles. (*b*) The APP is cleaved, producing β-amyloid (Aβ-42) fragments which are in an *α*-helix conformation. These change to a β-pleated sheet conformation, oligomerize, forming amyloid plaques.

Normal microtubules which serve as the pathways for anterograde and retrograde transport within the neuron unravel as the soluble monomeric tau proteins become hyperphosphorylated and detach from the microtubule. These hyperphosphorylated tau proteins form paired helical filaments, and thence aggregates leading to NFT (figure 4*a*). In the β-amyloid plaque pathway (figure 4*b*), amyloid precursor protein (APP) is cleaved by β-secretase and *γ*-secretase into β-amyloid fragments. Although the initial confirmation of Aβ-42 is an *α*-helix, transformation to a β-pleated sheet conformation is a necessary step in plaque formation. When the β-pleated sheets oligomerize, they can eventually join into polymers which form plaques (figure 4*b*). Our data suggest that chronic dietary exposure to BMAA triggers both the NFT and β-amyloid pathways.

### (c) Neuroprotective mechanisms of l-serine

Larger BMAA doses resulted in increased protein-bound BMAA concentrations in the brain. However, although l-serine reduced NFT density, it did not alter the ratio of protein-bound to total BMAA, which may be physiologically invariant. Possible neuroprotective mechanisms of l-serine include prevention of BMAA misincorporation in specific proteins involved in NFT formation. Misincorporation at rates as low as 1/10 000 can result in neurodegeneration [35], but such levels may be below our ability to differentiate. There may also be additional neuroprotective mechanisms other than prevention of misincorporation.

### (d) Implications of chronic BMAA exposure for neurodegenerative disease

BMAA-producing cyanobacteria occur globally, perhaps causing similar neuropathologies. Our finding that all of the low-dose vervets developed tauopathies with NFT has implications for human health. BMAA may serve as an environmental trigger for some forms of other neurodegenerative illnesses including sporadic ALS and AD. In human beings, increasing age is a risk factor for ALS, AD and PD. We have initiated experiments to determine if chronic dietary exposures of aged vervets to BMAA results in more profound histopathology.

We have sponsored FDA-approved human clinical trials (ClinicalTrials.gov Identifier NCT01835782) to determine if L-serine is a safe and efficacious treatment to reduce disease progression in ALS patients. We hope to initiate human clinical trials of L-serine for mild cognitive impairment and early onset AD in the near future.

In conclusion, Koch's postulates [38] have been satisfied with respect to establishing chronic dietary exposure to BMAA as a cause of a neurodegenerative illness: (i) BMAA has been identified in post-mortem brain tissue from ALS/PDC patients from Guam who consume a BMAA-rich diet but not in control patients who have not been exposed to the traditional Chamorro diet, (ii) vervets fed BMAA over 140 days developed NFT and β-amyloid deposits, and (iii) BMAA was isolated and identified in BMAA-fed vervets that had NFT and β-amyloid deposits in their brains. This study indicates that chronic exposure to BMAA can trigger neurodegenerative illness and that adding L-serine to the diet can reduce the risk of disease.

## Supplementary Material

SupplementaryMat - Cox et al -Dec2015
