# Supplementary material for: Dietary exposure to an environmental toxin triggers neurofibrillary tangles and amyloid deposits in the brain
Source: Proc Biol Sci. 2016 Jan 27;283(1823):20152397. doi: 10.1098/rspb.2015.2397 (PMC4795023; doi:10.1098/rspb.2015.2397)
Supplement: SupplementaryMat - Cox et al -Dec2015 [file rspb20152397supp1.pdf]

Supplementary Material Table 1.
Analytical Chemistry: Kruskal-Wallis (H-statistic calculated without including controls to avoid excess skewing to statisical significance.)

		Treatment	Median	H statistic
Plasma	Protein BMAA concentration (µg/ml)	Control	ND	5.56 NS
		Low Dose BMAA	0.91	
		High Dose BMAA	1.23	
		BMAA +Serine	2.16	
	(Protein/Total) * 100 BMAA concentration	Control	ND	13.24 **
		Low Dose BMAA	7.98	
		High Dose BMAA	8.40	
		BMAA +Serine	12.00	
Brain	Protein BMAA concentration (µg/mg)	Control	ND	8.69**
		Low Dose BMAA	0.32	
		High Dose BMAA	0.75	
		BMAA +Serine	0.83	
	(Protein/Total) * 100 BMAA concentration	Control	ND	0.05 NS
		Low Dose BMAA	4.65	
		High Dose BMAA	4.82	
		BMAA +Serine	4.72	
CSF	Total BMAA concentration (µg/ml)	Control	ND	9.09**
		Low Dose BMAA	0.26	
		High Dose BMAA	0.71	
		BMAA +Serine	0.58	

ND = not detected; NS = not significant ($p>0.05$)
** $p < 0.01$

Supplementary Material Table 2.
Median counts for density of AT8 IHC positive inclusions generated by NIH Image J64 software from TissueScope™LE instrument,
Jonckheere-Terpstra Trend Statistics (z statistic)

Anatomical Region	Control	Low Dose BMAA	BMAA +Serine	High Dose BMAA	z Statistic	p value
Temporopolar Cortex (Dorsal)	38	46	53	162	3.65	0.0001
Temporopolar Cortex (Ventral)	54	77	79	191	3.92	<0.0000
Occipital Cortex	43	65	124	136	4.55	<0.0000
Entorhinal Cortex (Anterior)	53	69	121	145	4.49	<0.0000
Superior Frontal Gyrus	19	58	95	106	4.59	<0.0000
Amygdala (Paralaminar Nucl.)	64	118	158	250	4.67	<0.0000
Perirhinal Cortex	61	79	101	165	4.97	<0.0000
Anterior Cingulate Gyrus	32	28	48	83	3.13	0.0009
Caudate Nucleus	10	1	29	30	3.16	0.0008
Substantia Nigra	9	5	38	35	3.97	<0.0000
Primary Motor Cortex	37	30	58	130	4.07	<0.0000
Dentate Gyrus	7	3	22	13	3.76	0.0001
Insula	43	33	54	153	3.73	0.0001
Entorhinal Cortex (Posterior)	57	106	85	178	4.87	<0.0000

Supplementary Material for Biochemical Analysis

Blinded samples were analysed following a validated, official method of BMAA analysis determined by the Association of Analytical Communities (AOAC International) LC-MS/MS method [43]. β-N-methylamino-L-alanine (BMAA) was compared to an authenticated standard (Sigma B-107 St. Louis, MO). L-2,4-diaminobutryic acid dihydrochloride (DAB-32830), β-N-methyl-d3-amino-DL-alanine-$^{15}N_2$, trichloroacetic acid (TCA, T6399), and ammonium acetate (Fluka 73594) were purchased from Sigma-Aldrich. Methanol (A456 Optima LC/MS) and Water (W-6 Optima LC/MS) were purchased from Fisher Scientific. Negative controls included matrix blanks from a control animal with no detectable BMAA, AQC derivatised blanks, internal standards, and solvent blanks (HCl, TCA). The TCA extract (prior to filtration) was hydrolysed for soluble protein in both plasma and brain samples, but since the amount of BMAA present was negligible, these samples were omitted from further analysis.

Frozen plasma samples were thawed and combined with an equal volume of cold 20% (w/v) TCA including an aliquot of internal standard (β -N-methyl-d3-amino-DL-alanine-$^{15}N_2$). Samples were precipitated at room temperature for 30 min and centrifuged at 14,000 x g for 5 min. The supernatant was removed and filtered using a centrifuge filter (0.2μm, Millipore UltrafreeMC) at 14,000 x g for 5 min. The filtered sample was diluted 1/50 with purified water (Millipore Direct Q-3uv, 18MΩ) and derivatised with 6-aminoquinolyl-N-hydroxysuccinimidyl carbamate (AQC, Waters AccQTag reagent, PN WAT052880) following steps to ensure a balanced reaction. To check for a balanced reaction, samples were derivatised at different dilutions and checked for linearity of signal and a preponderance of double derivatised lysine. The final derivatisation was 10 μl of the diluted 1/50 sample plus 70 μl borate buffer plus 20 μl AQC. This fraction is predominately free amino acids. Standards were prepared in control animal plasma matrix, wherein BMAA was not detected. Daily standard curves, prepared in both a free plasma matrix and a hydrolysed plasma matrix, were used for analyte

quantification. A comparison between standard curves prepared with and without a matrix was also completed and the variation between the two was negligible.

Following the removal of supernatant from the plasma, the remaining pellet was transferred to a glass vial using two washes of 6 M HCl totalling 390 µl. A 10 µl aliquot of internal standard was also added. Samples were heated at 110°C for 18 h and centrifuge filtered at 14,000 x g for 5 min. The filtered hydrolysate was diluted 1/50 with purified water (Millipore Direct Q-3uv, 18MΩ) and derivatised (8 µl diluted sample plus 72 µl borate buffer plus 20 µl AQC). This AQC derivatisation of protein-bound amino acids followed a step to check that a balanced reaction was achieved, as stated above. The concentration of the internal standard was within the same range as the concentration of BMAA within the samples.

Protein concentration was low within the cerebral spinal fluid (CSF) and due to limited sample, we quantified total BMAA in CSF. To measure the total concentration of BMAA in the CSF a subsample was transferred to a glass vial and diluted with an equal volume of 12 M HCl including an aliquot of internal standard. The sample was heated at 110°C for 18 hrs followed by filtering using a centrifuge filter as outlined above. The sample was then diluted 1/50 in purified water (Millipore Direct Q-3uv, 18MΩ) and derivatised with AQC (20 µl diluted sample plus 60 µl borate buffer plus 20 µl AQC) following a step to check that a balanced reaction was achieved, as stated above.

Frozen brain samples (50 mg) were sonicated on ice (2 watts 10 sec x 3) in cold 20% (w/v) TCA (90 µl) followed by the addition of an internal standard. Samples were left to precipitate overnight (3°C) and were subsequently centrifuged at 14,000 x g for 5 min. The supernatant was removed followed by an addition of a second aliquot (100 µl) of cold 20% (w/v) TCA and sonicated, as before. The sample was then left to rest at room temp for 2 hrs before centrifuging at 14,000 x g for 5 min. The supernatant was removed and pooled with the prior supernatant and centrifuge filtered (0.2 µm). The filtered supernatant was diluted 1/50 with

purified water (Millipore Direct Q-3uv, 18MΩ) as determined by a check for a proper AQC balanced reaction and derivatised (10 µl diluted sample + 70 µl borate buffer + 20 µl AQC). This fraction contains the free amino acids from the brain tissue.

Hydrochloric acid (190 µl of 6 M) was added to the brain protein pellet remaining after the supernatant was removed along with 10 µl of internal standard. The sample was heated at 110°C for 18 hrs and then centrifuged filtered (0.2 µm). The filtered sample was diluted 1/50 in purified water (Millipore Direct Q-3uv, 18 MΩ) as determined by ensuring that the AQC reaction was balanced and derivatised (10 µl diluted sample + 70 µl borate buffer + 20 µl AQC).

All samples were analysed on a TSQ Quantiva (Thermo Scientific) triple quadrupole mass spectrometer with an Ultra High Pressure Liquid Chromatography (Waters Acquity-UHPLC) system equipped with a Binary Solvent Manager, Sample Manager, and a Phenomenex Kinetex column (#00F-4475AN, 150 × 2.1 mm, 1.7 µm C18, 100A) at 65°C. Mobile phase A was 20 mM ammonium acetate (adjusted to pH 5.0 using glacial acetic acid) and mobile phase B was 100% methanol. Separation was achieved using an isocratic elution at 0.35 ml/min for 3.5 min followed by a 1.5 min wash phase and a one minute re-equilibration phase.

Nitrogen gas was supplied to the heated electrospray ionization (H-ESI) probe with a sheath gas pressure of 40 Arb, aux gas of 2 Arb, sweep gas of 1 Arb. Samples were analysed in positive ion mode with a vaporizer temperature of 400°C, capillary temperature of 350°C, and spray voltage 3500 V. The second quadrupole was pressurized to 1.0 mTorr with 100% argon. Product-ion analysis of BMAA used *m/z* 459 as the precursor ion for collision-induced dissociation (CID) and thereby all other ions were excluded in the first quadrupole. Further two-step mass filtering was performed during selective reaction monitoring (SRM) of BMAA after CID in the second quadrupole, monitoring the following transitions: *m/z* 459 to 119, CE 25 eV; 459 to 188 CE 38 eV; 459 to 214 CE 35 eV; 459 to 258 CE 36 eV; 459 to 289 CE 23

eV; 459 to 171 CE 45 eV. The resultant product ions were detected, after passing the third quadrupole and their relative abundances were quantified. Double ionized AQC derivatised BMAA was also monitored with a precursor ion of *m/z* 230 and a product ion of 171 CE 27 eV. Additionally, the following amino acids were monitored as both a parent ion (CE 0 eV) and with a transition to the product ion 171 CE 21: single derivatised lysine *m/z* 317, double derviatised lysine *m/z* 487, leucine *m/z* 302, serine *m/z* 276. The internal standard (β-N-methyl-d3-amino-DL-alanine-^{15}N2) with a precursor ion of *m/z* 464 and product ions *m/z* 124 CE 25 eV, *m/z* 171 CE 45, *m/z* 259 CE 36, and *m/z* 294 CE 23 was also monitored.

BMAA tissue concentrations were determined relative to concentration curves run daily in spiked matrix samples from a control animal. All samples and spiked samples had an internal standard of β-N-methyl-d3-amino-DL-alanine-$^{15}N_2$. Ruggedness (intermediate precision) was calculated from standards prepared in a control matrix, injected 3-5 times per day and analysed from iterative preparations over a five day period. Repeatability was determined from a single spiked matrix sample run ten times in a single day. Recovery was calculated from low concentration BMAA spikes into a blood plasma matrix of a control animal with no detectable BMAA. Detection limits (LOD) and limits of quantification (LOQ) of BMAA were determined experimentally by injecting a dilution series of authenticated stock solutions spiked into a blood plasma matrix injected in triplicate at 8 concentrations (0, 0.001, 0.005, 0.01, 0.05, 0.1, 0.2, 1 µg/ml). Linear response was calculated as R^2= 99.9%. LOD and LOQ were estimated following EPA guidelines (n=7; LOD = $t_{0.99}$*S; LOQ = 3*LOD; where t= one-tailed t-statistic at the 99% confidence level for n-1 replicates and S= standard deviation of n samples spikes at the estimated LOQ).

Repeatability and ruggedness had a RSD of 2% and 3% respectively. Recovery mean was 104% (range = 95-117%, RSD =8%) with good signal to noise (range 68-10,311). LOD and LOQ were measured at 3.2 and 9.6 ng/ml respectively.
